# 55. Infective Endocarditis After Surgical or Transcatheter Aortic Valve Replacement

**DOI:** 10.1093/ofid/ofab466.055

**Published:** 2021-12-04

**Authors:** Aikaterini Papamanoli, Brandon Muncan, Puja Parikh, Hal A Skopicki, Andreas Kalogeropoulos

**Affiliations:** Stony Brook University Hospital, Stony Brook, New York

## Abstract

**Background:**

Infective endocarditis (IE) can complicate both surgical aortic valve replacement (SAVR) and transcatheter aortic valve implantation (TAVI) with significant morbidity and mortality despite differing pathogenesis. In the presence of limited data from direct comparison studies and recent expansion of TAVI to younger and lower- risk patients, we compared the incidence and timing of IE in TAVI versus SAVR.

**Methods:**

Using data from the TriNetX electronic health records network, we identified (1) a cohort of patients who underwent TAVI between January 2016 and December 2020 (CPT procedure code 1021150) and (2) a propensity score-matched cohort of patients who underwent SAVR (CPT procedure codes 1035167 or 1029693, without any associated transcatheter procedure). We examined the incidence of IE (captured with ICD-10 codes I33, I38, or I39) over a 5-year follow up period and matched the cohorts for demographic data and clinically relevant background history. We used Kaplan-Meier estimates and Cox proportional hazards models to compare incidence between matched cohorts.

**Results:**

We identified 6,302 patients with TAVI and 6,302 matched patients with SAVR. The baseline characteristics of the cohorts were well balanced, **Table 1**. All standardized mean differences were < 0.05, indicating adequate matching between cohorts. The Kaplan-Meier mortality at 5 years was 38.0% in the TAVI vs. 22.0% in the SAVR cohort (log-rank P < 0.001). There were 290 cases with IE in the TAVI and 604 cases in the SAVR cohort. The corresponding 5-year event rates were 10.0% vs. 16.9% (log-rank P < 0.001), respectively, **Figure 1.** The risk ratio of TAVI vs. SAVR related IE over the entire 5-year period was 0.48 (95%CI 0.42 — 0.55; P < 0.001). However, the relative risk for IE was non-proportional between groups over the 5-year period, with an early pronounced incidence among SAVR relative to TAVI patients and gradual convergence of the hazard rates over time, **Figure 2.**

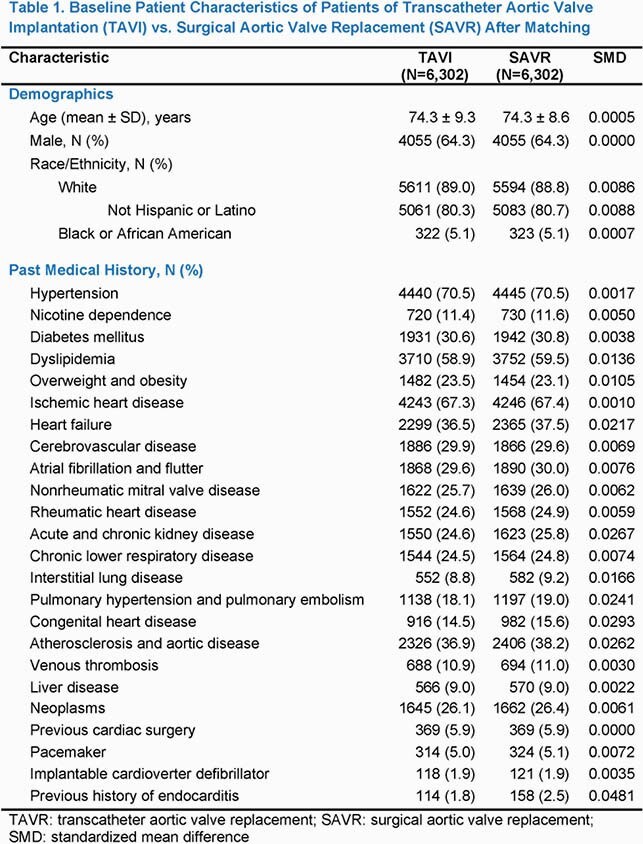

Figure 1. Cumulative 5-Year Incidence (Kaplan-Meier Estimates) of Infective Endocarditis Among Matched Transcatheter Aortic Valve Implantation (TAVI) vs. Surgical Aortic Valve Replacement (SAVR) Recipients

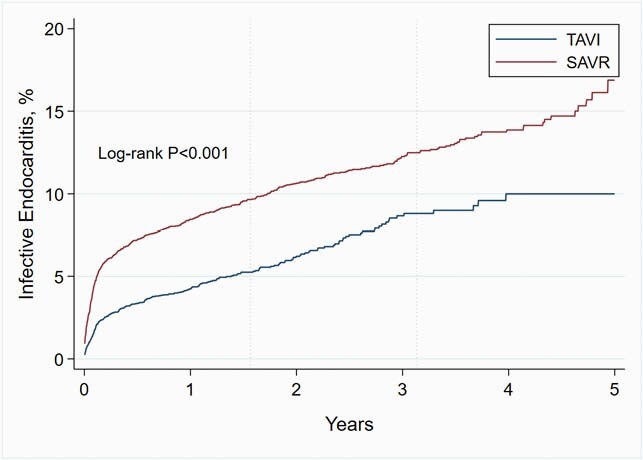

Figure 2. Risk of Infective Endocarditis in SAVR vs. TAVI Recipients Over Time

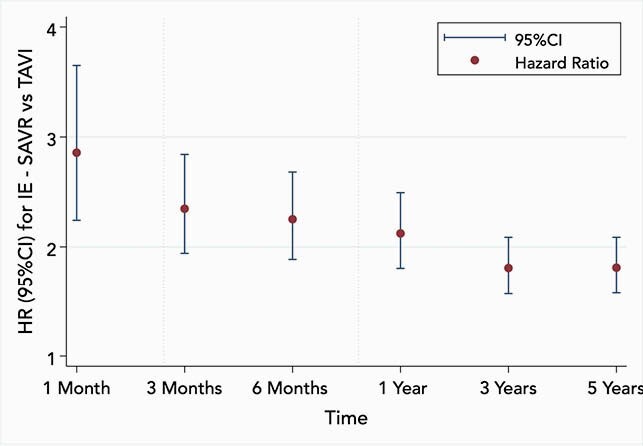

**Conclusion:**

In this comparative study, the risk for IE was lower among TAVI vs. SAVR recipients, primarily due to the higher risk of IE during the early post-SAVR period. With increasing uptake of TAVI procedures, a better understanding of the temporal occurrence and pathophysiology of IE and application of effective treatment strategies in these patients is required.

**Disclosures:**

**All Authors**: No reported disclosures

